# Changes in Polar Lipid Composition in Balsam Fir during Seasonal Cold Acclimation and Relationship to Needle Abscission

**DOI:** 10.3390/ijms242115702

**Published:** 2023-10-28

**Authors:** Mason T. MacDonald, Rajasekaran R. Lada, Gaye E. MacDonald, Claude D. Caldwell, Chibuike C. Udenigwe

**Affiliations:** 1 Department of Plant, Food, and Environmental Sciences, Faculty of Agriculture, Dalhousie University, Bible Hill, NS B2N 5E3, Canada; raj.lada@dal.ca (R.R.L.); gemacdon@dal.ca (G.E.M.); claude.caldwell@dal.ca (C.D.C.); 2 School of Nutritional Sciences, Faculty of Health Sciences, University of Ottawa, Ottawa, ON K1N 6N5, Canada; cudenigw@uottawa.ca

**Keywords:** *Abies balsamea*, cold, conifer, fluorescence, galactosyldiacylglycerol, galactolipids, phospholipids, membrane injury, needle retention

## Abstract

Needle abscission in balsam fir has been linked to both cold acclimation and changes in lipid composition. The overall objective of this research is to uncover lipid changes in balsam fir during cold acclimation and link those changes with postharvest abscission. Branches were collected monthly from September to December and were assessed for cold tolerance via membrane leakage and chlorophyll fluorescence changes at −5, −15, −25, −35, and −45 °C. Lipids were extracted and analyzed using mass spectrometry while postharvest needle abscission was determined gravimetrically. Cold tolerance and needle retention each significantly (*p* < 0.001) improved throughout autumn in balsam fir. There were concurrent increases in DGDG, PC, PG, PE, and PA throughout autumn as well as a decrease in MGDG. Those same lipids were strongly related to cold tolerance, though MGDG had the strongest relationship (R^2^ = 55.0% and 42.7% from membrane injury and chlorophyll fluorescence, respectively). There was a similar, albeit weaker, relationship between MGDG:DGDG and needle retention (R^2^ = 24.3%). Generally, a decrease in MGDG:DGDG ratio resulted in better cold tolerance and higher needle retention in balsam fir, possibly due to increased membrane stability. This study confirms the degree of cold acclimation in Nova Scotian balsam fir and presents practical significance to industry by identifying the timing of peak needle retention. It is suggested that MGDG:DGDG might be a beneficial tool for screening balsam fir genotypes with higher needle retention characteristics.

## 1. Introduction

Balsam fir is a mid- to late-successional conifer species native to northeastern United States and Canada [1]. In Canada, balsam fir is found as far east as Newfoundland and as far west as Alberta, though balsam fir stands are more scattered and not the dominant conifer in most western forests [2]. Balsam fir can be used for lumber or fuel, but they are also commonly used as Christmas trees. Balsam fir Christmas trees are often harvested in early October to meet export demand and are often a preferred species due to their unique fragrance, color, and high needle retention characteristics [3]. However, postharvest needle retention has decreased over time, attributed to earlier harvests and climate change limiting opportunities for cold acclimation [4].

Optimum growth of balsam fir occurs in regions with an annual temperature of 2 to 4 °C. Ideally the coldest winter temperatures would range from −18 to −12 °C, while the warmest summer temperatures would range from 16 to 18 °C [2]. Few areas in Canada provide an ideal environment for balsam fir. As an example, Nova Scotia has the warmest average temperature of any Canadian province, with an average temperature of 25.9 °C in July 2023 [5]. Yet winter temperatures the same year fell as low as −26 °C [5]. There have been fewer frost events or cold degree days in autumn in which plants could acclimate for winter conditions, possibly exacerbating the opportunity for cold stress during winter.

Cold stress and/or freezing temperatures can cause widespread metabolic dysfunction in plants, including reduced electron transport, increased oxidative stress, impaired water movement, changes in membrane fluidity, and eventual tissue death [6,7]. Like many conifers, balsam fir is adept at tolerating freezing temperatures through cold acclimation. Cold acclimation is regulated by a complex network of signaling pathways triggered by environmental cues like light and temperature [8]. Decreasing temperatures and shorter photoperiods trigger cryoprotective genes that ultimately improve protein stabilization, increase solute concentrations, and alter membrane composition [9,10].

Cold acclimation-induced membrane changes are manifested through shifts in the concentration of membrane lipids. One change is that the concentration of monogalactosyldiacylglycerol (MGDG) tends to decrease in tandem with an increase in digalactosyldiacylglycerol (DGDG) [11,12]. MGDG forms a single layer of lipids for greater efficiency of thylakoid membranes while DGDG forms a bilayer to create greater stability [13]. A shift towards DGDG constitutes a shift towards membrane stability in the plant and helps protect plants from cold stress. A second shift towards phospholipids (PLs) occurs after cold acclimation [14]. More specifically, there is an increase in phosphatidycholine (PC) and phoshatidylethanolamine (PE) after cold acclimation [12,15]. An increase in PLs is associated with a shift towards lipids containing unsaturated fatty acids to help maintain membrane fluidity in cold temperatures [16].

Cold acclimation and tolerance can be calculated through many different methods, but in essence all methods operate by exposing plants to cold temperatures and assessing damage [17]. The specific temperatures, exposure time, and damage assessment vary between studies and plant species [17]. One technique is to assess plants for visual damage from freezing temperatures, though this can often be a tedious task [17]. Alternatives include measuring electrolyte leakage [18,19] or chlorophyll fluorescence [20,21] to assess damage. Freezing tolerance is then often quantified using an LT50 value, or the temperature at which there is 50% electrolyte leakage or a 50% decrease in chlorophyll fluorescence. Freezing tolerance of *Abies* species ranged from −25 to −70 °C through visual observation [22]. Freezing tolerance of *Abies procera* was monitored throughout autumn and ranged from approximately −20 °C in September to −38 °C in December [23].

Cold acclimation tends to be associated with improved postharvest needle retention in balsam fir trees. Needle retention increases throughout the autumn months and reaches a peak in November or December [3]. Though improved needle retention was strongly correlated with decreasing temperatures and photoperiod, freezing tolerance of balsam fir has not been directly related to needle retention. Further, postharvest needle retention is linked to changes in lipids and fatty acids [24]. Abscising needles had lower concentrations of MGDG and DGDG, but significantly higher concentrations of PC, lysophosphatidylglycerol (LPG), and phosphainositol (PI) than intact needles [24]. It is reasonable to postulate that lipid changes in balsam fir are linked to both cold tolerance and postharvest needle retention. The objectives of this study are to (1) quantify changes in balsam fir cold tolerance throughout autumn, (2) determine changes in balsam fir needle polar lipids throughout autumn, and (3) to relate cold tolerance to changes in lipid concentration and needle retention in balsam fir. 

## 2. Results

### 2.1. Confirming Cold Acclimation

**Figure 1:**
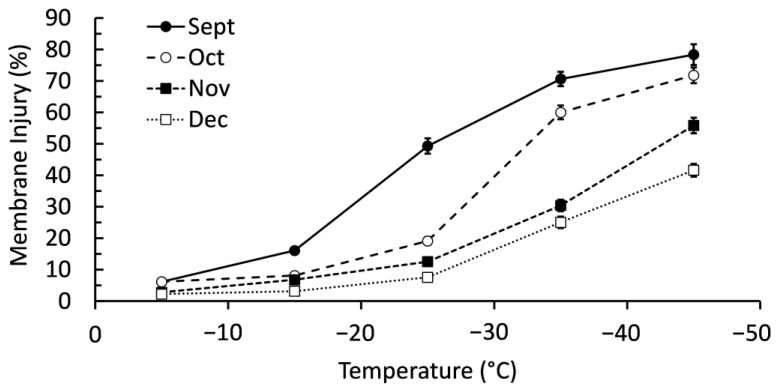
Membrane injury of balsam fir branches collected from September to December then subjected to freeze tests at −5, −15, −25, −35, and −45 °C. Each data point is the mean ± standard error as calculated from 20 replicates.

There was a significant interactive effect (*p* < 0.001) between collection month and freezing temperatures on membrane injury (Figure 1). Branches collected in September had significantly more membrane injury once exposed to −15 °C, a trend that continued until exposure to −45 °C when there was no difference between branches collected in September and November. Exposure to −25 °C was the point where there was very clear separation in membrane injury between sample months; highest to lowest membrane injury occurred in the order of September, October, November, and December. Membrane injury was higher in all collection months when exposed to −35 °C, though the order was identical to −25 °C freezing. Ultimately, there was a clear trend of balsam fir having lower membrane injury in cold temperatures when harvested later in autumn. Blocking by genotype caused minimal improvement to the statistical model (F = 0.94, *p* = 0.420).

**Figure 2:**
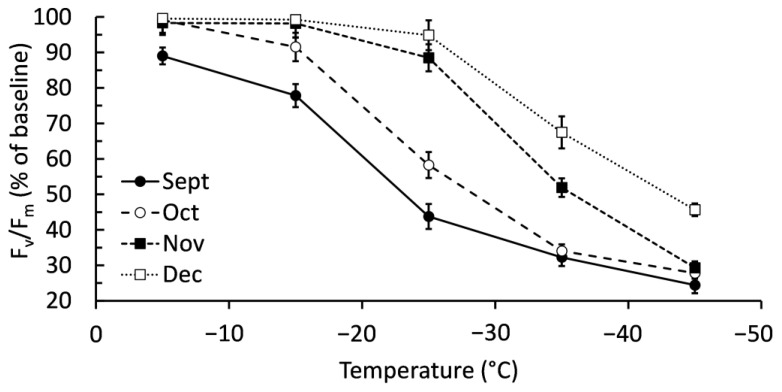
Chlorophyll fluorescence (F_v_/F_m_) of balsam fir branches collected from September to December and then subjected to freeze tests at −5, −15, −25, −35, and −45 °C. Chlorophyll fluorescence is expressed as a percentage of the baseline values established by measuring branches that were not exposed to any freezing test. Each data point represents the mean ± standard error as calculated from 20 replicates.

There was a significant interactive effect (*p* < 0.001) between collection month and freezing temperatures on chlorophyll fluorescence (Figure 2). September had significantly lower fluorescence than other months when exposed to −5 °C, though fluorescence was still maintained at approximately 90% of the baseline value. September was also the only sampling month with significantly lower fluorescence after −15 °C exposure. Both September and October had significantly lower fluorescence after exposure to −25 °C. December maintained significantly higher fluorescence than other months after exposure to −35 °C and −45 °C, though fluorescence had decreased compared to freeze tests at −5, −15, and −25 °C. As with membrane injury, the general trend was balsam fir maintaining their chlorophyll fluorescence in cold temperatures when harvested later in autumn. Blocking by genotype improved the statistical model with respect to chlorophyll fluorescence (F = 3.57, *p* = 0.014).

**Figure 3:**
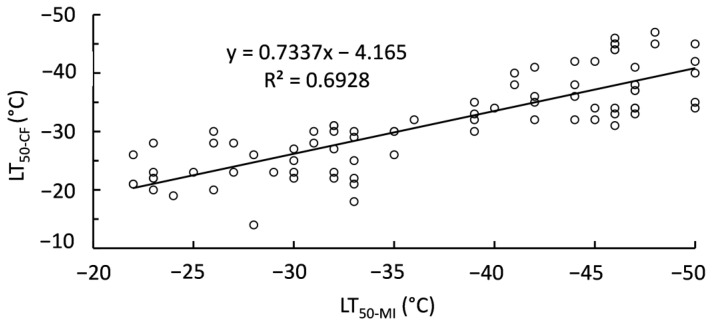
Linear relationship between LT50-MI and LT50-CF (N = 80).

**Figure 4:**
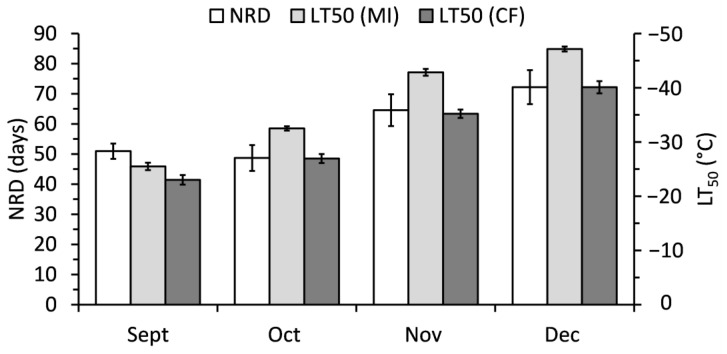
NRD and LT50 values estimated from membrane injury (LT50-MI) and chlorophyll fluorescence (LT50-CF) from balsam fir branches collected in September to December. Bars represent the mean ± standard error as calculated from 20 replicates.

Sigmoidal curves fitted to membrane injury and chlorophyll fluorescence responses to freezing temperatures allowed for the determination of LT50 values. However, LT50 values were not completely consistent between LT50 calculated from membrane injury (LT50-MI) and chlorophyll fluorescence (LT50-CF) (Figure 3). A perfect relationship between LT50-MI and LT50-CF would have a slope of 1 compared to the calculated 0.7337. Instead, LT50-MI values are lower than their respective LT50-CG estimates. Sampling month had a consistent significant (*p* < 0.001) effect on LT50, regardless of estimation method. September always had the highest LT50, while December always had the lowest LT50 (Figure 4).

Sampling month had a significant (*p* < 0.001) effect on needle retention duration (NRD) (Figure 4). September and October had the lowest NRD of 51 and 49 days, respectively. November and December had a 32–47% increase in NRD compared to September and October, though there was no significant difference between November and December. Although the overall trend was consistent with LT50 values per month, NRD was only weakly correlated to LT50-MI and LT50-CF (r = −0.365 and −0.290, respectively). Genotype contributed significant variation to NRD (F = 8.90, *p* < 0.001). A regression equation using genotype as a block improved the relationship between NRD and LT50-MI (R^2^ = 35%) and between NRD and LT50-CF (R^2^ = 36%).

### 2.2. Changes in Polar Lipids during Cold Acclimation

Sampling month had a significant effect (*p* < 0.05) on all polar lipid classes in balsam fir except for LPG and LPE (Table 1). Most polar lipids increased in relative concentration throughout autumn. There was a relative increase of 11.8% in DGDG, 30.3% in PC, 26.5% in PG, 81.7% in PE, and 23.1% in PI from September to December. The increases in the 5 lipid classes above were offset by a 26.2% decrease in MGDG over the same time span. LPG and PA did not have a consistent progression from September to December; instead, each reached their highest relative concentration in November before decreasing in December.

**Figure 5:**
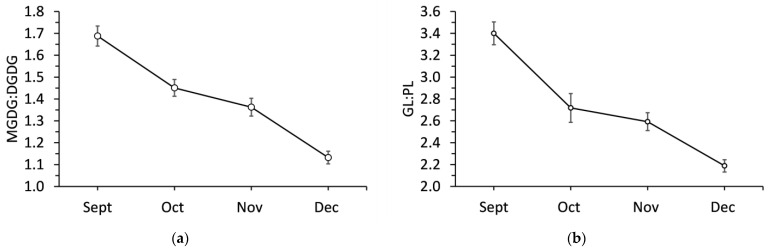
Ratio of (**a**) monogalactosyldiacylglycerol to digalactosyldiacylglycerol and (**b**) galactolipids to phospholipids in balsam fir collected from September to December. Each data point represents the mean ± standard error as calculated from 20 replicates.

Sampling month had a significant effect (*p* < 0.001) on MGDG:DGDG and GL:PL ratios (Figure 5). The MGDG:DGDG was 1.69 in September and then significantly decreased each month. MGDG:DGDG decreased by 14.2% in October, 19.5% in November, and 33.1% in December all compared to September. GL:PL significantly decreased by 20.0% from September to October. There was a further significant decrease in November, but by December, GL:PL had decreased by 35.6% compared to its initial value.

### 2.3. Relationship of Polar Lipids and Lethal Temperatures

Almost all polar lipid classes had significant (*p* < 0.05), linear relationships with LT50 values, except for PA, LPC, LPG, and LPE (Table 2). MGDG had the strongest relationship with LT50, accounting for 55.0% of variation in LT50-MI and 42.7% of variation in LT50-CF. MGDG also had the only positive relationship with LT50 values. DGDG, PC, PE, and PI were weaker, accounting for 30.0–36.7% of the variation in LT50-MI and 22.9–29.5% of the variation in LT50-CF. PG accounted for less than 20% of the variation in each LT50 value.

**Figure 6:**
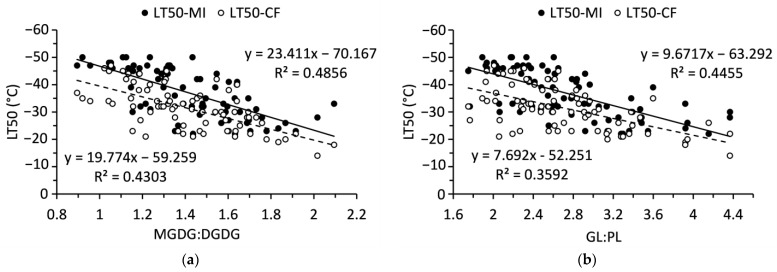
Relationships of (**a**) monogalactosyldiacylglycerol to digalactosyldiacylglycerol ratio and (**b**) galactolipid to phospholipid ratio to LT50 values in balsam fir collected from September to December. N = 80.

MGDG:DGDG and GL:PL ratios each had significant, linear relationship with LT50 values (Figure 6). These relationships were stronger when LT50 was determined from membrane injury as opposed to chlorophyll fluorescence. The slope of each relationship was positive so that a shift towards DGDGs or PLs was related to lower LT50.

### 2.4. Relationship of Polar Lipids and Needle Abscission

**Figure 7:**
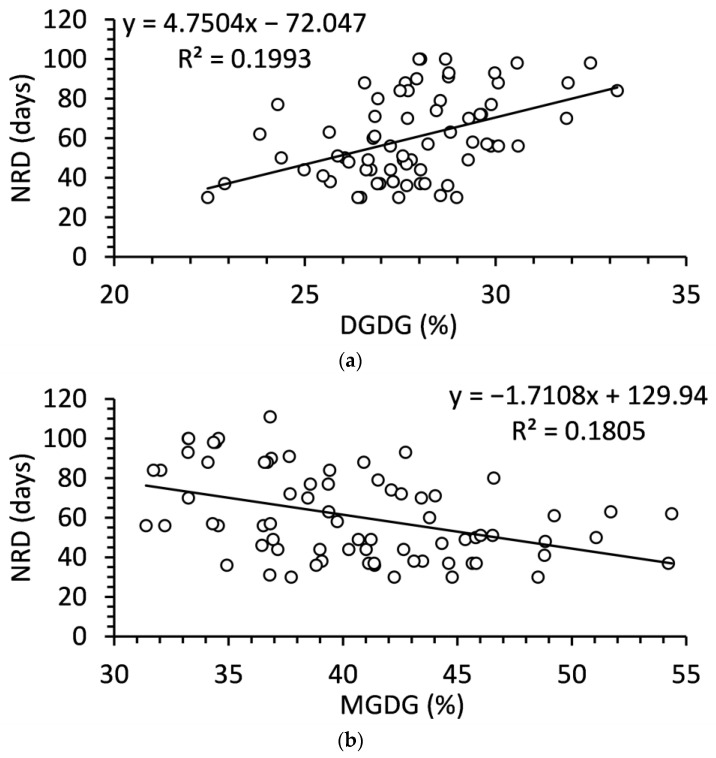

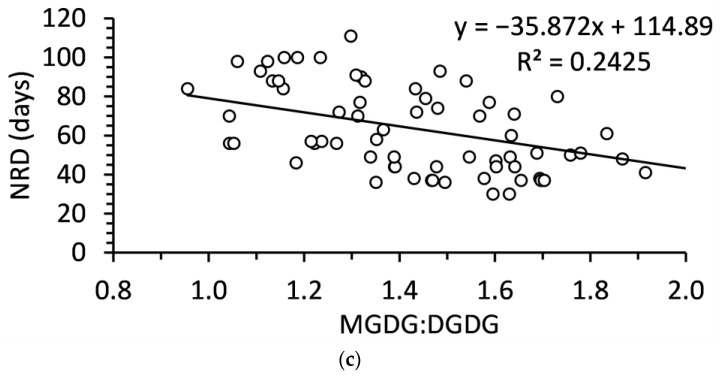
Relationships of (**a**) digalactosyldiacylglycerol, (**b**) monogalactosyldiacylglycerol, and (**c**) monogalactosyldiacylglycerol to digalactosyldiacylglycerol ratio to needle retention duration (NRD) in balsam fir collected from September to December. N = 80 in each.

Only DGDG and MGDG were significantly related to NRD (Figure 7). DGDG was positively related to NRD while MGDG was negatively related to NRD. Although each of the relationships was significant, these were relatively weak relationships that accounted for less than 20% of the variation in NRD. The MGDG:DGDG ratio slightly improved the model, accounting for 24% of the variation in NRD (Figure 7).

## 3. Discussion

### 3.1. Cold Acclimation in Balsam Fir

There is sufficient evidence to support that balsam fir acclimates to cold temperatures throughout autumn in Nova Scotia. Balsam fir branches collected each month after September better resisted membrane injury and impaired chlorophyll fluorescence when subjected to freezing temperatures. The LT50 value for branches collected in December was between −40 and −50 °C, almost twice as low as branches collected in September. Freezing tolerance of balsam fir fall well within the expected range of −25 to −70 °C for *Abies* species [22].

The fact that balsam fir cold acclimates through autumn was expected. The minimum temperature observed in December during sampling was −23 °C and average temperatures continued to decrease in January and February. Although rare, it is possible to have minimum temperatures below −40 °C in Nova Scotia where these branches were collected. Balsam fir would need to adapt to such freezing temperatures to survive the winter. However, it was important to confirm and quantify their ability to acclimate to freezing temperatures. One weakness of previous cold acclimation studies in balsam fir was that it was assumed that cold acclimation occurred, but the degree of that acclimation was not measured. The LT50 values in this current study lend credence that the assumption of cold acclimation was likely correct in previous studies. It is noteworthy that accuracy of estimates in this current study could be improved with smaller increments in freezing temperatures tested [17]. Also, a few branches from December did not have 50% membrane injury or a 50% decrease in chlorophyll fluorescence even at −45 °C, although they were usually very close to that 50% mark. In those cases, LT50 values were extrapolated from a sigmoidal curve. Future evaluations of balsam fir freezing tolerance should include lower temperatures to avoid that problem.

### 3.2. Polar Lipids in Balsam Fir

Seasonal changes in lipid profiles have been studied extensively in other species, but there have been few studies of lipids in balsam fir. There are fewer major contributing spe-cies of polar lipids in balsam fir needles than in Arabidopsis rosettes [25]. GLs are the most abundant lipid classes in balsam fir needles, which can represent up to 80% of all polar lipids [26]. In balsam fir, galactolipids (GLs) comprise 65–70 per cent of all polar lipids. The other major contributor was PC and to a lesser extent PG. A previous study found 33% MGDG and 28% DGDG in balsam fir harvested in December [24]. Our study found 33% MGDG to 30% DGDG, resulting in a very similar ratio as previous work [24]. This ratio is higher than reported in most plants, but that could be because plants in both studies were subjected to cold acclimation [25].

The seasonal changes in balsam fir GLs are consistent with those in Pinus sylvestris [27]. MGDG decreased during the winter months and increased in the spring, while DGDG increased. When membranes of the chloroplast were isolated from rye leaves, there was a decrease in MGDG and increase in DGDG due to cold acclimation [28]. Similar re-sults were observed in Arabidopsis [25]. Studies have shown that MGDG, DGDG, sulfoqui-novosyl diacylglycerol (SQDG), and PG are all involved in maintaining the thylakoid structure and for the proper functioning of photosystem II and related proteins [11,12]. Therefore, changes in GLs were expected during cold acclimation. 

MGDG and DGDG are also known to stabilize the photosystem protein complexes in chloroplasts [29]. Maintaining a constant MGDG:DGDG ratio in thylakoid membranes (at least under standard growth conditions) seems crucial for the stability and functional integrity of photosynthetic membranes [26]. An increase in the bilayer-forming galactolipid, DGDG, and a decrease in the monolayer-forming MGDG during cold acclimation should help protect the chloroplast membranes from damage during the winter keeping the membrane more fluid [26]. Due to the reduction of light and colder temperatures associated with winter, chloroplast membrane stabilization becomes ultimately important for survival. The ratio of MGDG to DGDG may vary according to the length of cold acclimation, as the biosynthesis of these compounds is tightly regulated to meet the needs of the cell under changing environmental conditions [25]. MGDG content decreased more than DGDG increased in this study. This can be explained by the fact that it takes two MGDG to form one DGDG [30]. The transition of MGDG to DGDG to maintain the appropriate ratio, which allows the reversible transition from the hexagonal II to lamellar α phase of the lipid bilayer, could be a very important factor in thylakoid biogenesis [31].

Most PLs increased during balsam fir cold acclimation, with the largest increase observed in PC. An increase in the per cent PC during cold acclimation has been reported in two other conifer species, *Pinus silvestris* and *Pinus nigra* [27,32]. As MGDG combines to form DGDG, the proportion of GL decreases while PL increases. With respect to cold acclimation, PLs are often of interest due to shifts towards longer and unsaturated fatty acid chains [28,33]. 

Although PC was the predominant PL, the relative increase in PLs was also a function of PG, PE, and PI. PI and PA have been identified to play a role in cell signaling [34]. PA is closely related to an increased activity of phospholipase D (PLD), which catalyzes the hydrolysis of PC to PA [35]. PA in balsam fir doubled from September to October but remained a minor contributor to overall PL concentration. Some studies with evidence of PLD activity have shown an increase from below 1 to 12 % in PA in a short time [25]. The increase in PA never exceeded 1% in balsam fir. Other PLs have been linked to a variety of stress responses. For instance, PI is the first molecule in the phosphoinositide signaling pathway [36] and increases have also been observed in drought and salinity stress [37]. The exact role of each PL in response to stress signaling needs to be further explored in plants, including balsam fir.

### 3.3. Needle Retention in Balsam Fir

Needle retention increased throughout autumn with peak retention occurring in November and December, consistent with several previous studies [38,39]. Previous studies had speculated that the improvement in needle retention was due to cold acclimation. This current study supports the hypothesis that cold acclimation improves needle retention through a significant relationship between LT50 values and needle retention. However, the relationship between cold acclimation and needle retention was weak unless genotype was considered. Needle retention is highly variable between genotypes [39], which at one point was attributed to differences in the rate at which certain genotypes acclimated to cold temperatures [39]. Even when genotype was included in the model, the model only explained 35–36% of the variation meaning there are likely other factors significantly affecting needle retention.

A decrease in MGDG and increase in DGDG were both associated with higher needle retention in balsam fir. As with cold acclimation in general, it is possible that increased stability of membranes due to higher proportion of DGDG also protects the plant from other stresses [26]. Membrane damage occurs prior to and during abscission in balsam fir, likely as a stress response postharvest [24]. Thus, any mechanism that protects membrane integrity might delay abscission. Although, the relationship between MGDG:DGDG ratio and needle retention was the strongest, it still only accounted for 24% of the variation. While shifting from MGDG to DGDG seems beneficial for needle retention, there must be other factors involved. 

Only accounting for a portion of variation in needle retention should not diminish the importance of cold acclimation and the shift towards DGDG. Abscission of balsam fir, like other plants, is a complex physiological phenomenon. Multiple factors have been associated with abscission, with the prevailing theory being that water stress is the impetus for postharvest needle abscission [3]. Synthesis of abscisic acid and ethylene occur postharvest, transpiration and water uptake decrease, and abscission occurs [3]. Meanwhile, having a high concentration of indole-3-acetic acid can delay abscission in balsam fir. Additional factors affecting balsam fir abscission include water quality, volatiles, nutrition, and others [3,4]. With such a multitude of other factors known to influence abscission, it makes sense that a shift in polar lipids is a part of the larger puzzle.

Cold acclimation could also affect postharvest abscission through other mechanisms than polar lipid shifts. Balsam fir accumulate carbohydrates and isopentenyladenosine during autumn, which were both associated with improved needle retention [3]. Other plants have shown a variety of cold acclimation specific proteins [8,40], which have never been assessed in balsam fir but could be linked to needle retention. There is also an established link between ethylene and cold acclimation in other species. Ethylene was synthesized during exposure to cold and upregulated cold acclimation in *Arabidopsis*; plants that could not synthesize adequate amounts of ethylene did not acclimate as well as those that could [41]. Where short-term exposure to ethylene helped promote cold tolerance in *Arabidopsis*, short-term ethylene exposure delayed abscission in balsam fir [3]. Any link between cold acclimation, ethylene, and needle abscission in balsam fir remains to be explored.

Furthering our understanding of the relationships between cold acclimation, lipid composition, and needle retention could have practical significance. First, the balsam fir industry has long desired screening tools to identify trees with superior needle retention. Previous studies identified ethylene evolution rates and sensitivity as one potential screening tool [3]. However, the study was limited by only uncovering differences between genotypes categorized as having low or high needle retention. The strength of the relationship between needle retention and ethylene sensitivity was not quantified. Second, there is the possibility of artificially inducing cold tolerance to promote needle retention. Antioxidant enzymes, certain hormones, and signaling metabolites all may modify local and systemic cold tolerance [42], thus may be useful to support high needle retention. Finally, it must be noted that lipidomics is only one strategy for understanding changes in physiology. Incorporation of other omic approaches, such as proteomics, genomics, transciptomics, and others would further our understanding.

## 4. Materials and Methods

### 4.1. Sampling and Experimental Design

The plant material for this investigation was collected from the Debert clonal tree orchard (45°44′ N, −63°50′ W) located in Debert, NS, Canada from September to January. Sampling month was an explanatory variable, with specific sampling dates being 18 September, 28 October, 25 November, and 30 December. The Christmas tree germplasm collection has over 220 genotypes of balsam fir. Previous research demonstrated considerable variation in needle retention between genotypes [39], so genotype was used as a blocking factor. Four genotypes were selected for levels of the blocking factor. The general experimental design included an explanatory variable with 4 levels, block with 4 levels, and was then replicated 5 times.

Branches to be sampled were identified on 20-year-old trees at an elevation of approximately 1 m from ground level on the south facing side of the trees. Branches with any visual problems, such as pathogen or pests, were avoided. Approximately 4 g of needles were removed onsite from each tree to be used for initial lipid analysis. Needle samples that were taken on site were immediately immersed in liquid nitrogen and stored at –80 °C until analysis. Branches were then cut to include the previous 2 years growth and taken to the lab for cold tolerance, needle retention, and eventual lipid analysis. Each month required 80 branches to be collected to be submitted to freeze testing, 20 branches to be assessed for needle retention, and 80 branches to be evaluated for lipid composition.

### 4.2. Environmental Conditions during Sampling Months

Temperatures and photoperiod from 30 days prior to the start of the experiment were recorded from the Debert Weather Station. Photoperiod was taken on the days of sampling only. However, temperatures leading up to the sampling days were used to calculate minimum temperature (T_min_), maximum temperature (T_max_), days of exposure to freezing temperatures, and cold degree days (CDD), which are all shown in Table 3. CDD was determined by using the following formula:

(1) $$CDD=\sum_{i=1}^{i=n}T_{base}-T_{a},ifT_{base}-T_{a}>0;otherwise\;0$$

In the above equation, T_a_ is the daily mean air temperature calculated from the daily minimum and maximum temperatures. T_base_ is the threshold temperature of 5 °C determined as the temperature at which plant growth no longer occurs in balsam fir [43], and i is day of the period prior to sampling, with 1 being the first day and n being the last day.

### 4.3. Display Conditions and Needle Retention

Branches were transferred to an environmental controlled growth chamber at a constant temperature of 20 °C and light intensity of 100µmol m-2 s-1. Light was supplied as a combination of incandescent and fluorescent. Both temperature and light conditions were selected to simulate household conditions as described by [44]. Once in the lab, branches were given a fresh aseptic cut 2.5 cm above the previous cut while submerged in water to reduce risk of cavitation. Branches were placed into amber bottles and provided 100 mL of distilled water. The neck of each flask was plugged with cotton wool to reduce direct water evaporation and provide added stability to a branch.

Needle retention was evaluated by lightly holding the branch between thumb and index finger and sliding along the length of the branch. Care was taken to slide in the same direction as the needles were attached to the stem. The goal was to dislodge needles that had abscised and not to snap off needles that were healthy. Needles were dried at 80 °C overnight and then weighed. This procedure was repeated every 2 days until complete needle shed. The day on which a branch lost 100% of its needles was referred to as its NRD.

### 4.4. Freeze Testing to Evaluate Cold Tolerance

Cold tolerance was assessed at 5 temperatures: −5, −15, −25, −35, and −45 °C. The temperature range was determined partially by the typical range of regional winter temperatures as well as cold tolerance estimates from other *Abies* species [22,23]. The freeze test protocol was based on previous work by [45]. Branches were placed in a programmable freezer (Thermotron SM-32-C, Holland, MI, USA), and the temperature was reduced 5 °C h^−1^ until reaching the target temperature. Branches were exposed to the target temperatures for 30 min, held at 0 °C overnight, and then returned to the growth chamber until the needles reached 20 °C for the evaluation of membrane injury and chlorophyll fluorescence.

Chlorophyll fluorescence (F_v_/F_m_), was determined using a MINI-PAM-II Photosynthesis Yield Analyzer (Heinz Walz, Effeltrich, Germany). Plants were dark-adapted for 20 min before fluorescence measurements with a saturating light pulse of 8000 µmol m^−2^ s^−1^. Three measurements were taken for each experimental unit; the average of these measurements was recorded as the F_v_/F_m_ for each branch. Chlorophyll fluorescence was measured prior to freezing test to establish a baseline for fluorescence in each branch. Chlorophyll fluorescence was measured again after the freezing test.

Membrane injury was determined from the percentage of electrolytes leaked into the solution versus the total electrolytes present. Test tubes were filled with 30 mL of distilled water and were allowed to adjust to room temperature (20 °C). The electrical conductivity of the distilled water (EC_w_) alone was measured using a CDM 2e Conductivity Meter (Bach-Simpson, London, ON, Canada). Afterward, approximately 0.4 g of needles were removed from each branch and completely submerged in a centrifuge tube. The tubes were sealed and left at room temperature for 24 h. Initial conductivity (EC_0_) was measured in order to determine electrolytes leaching into solution. Sealed tubes were then placed in a forced-air oven for 4 h at 90 °C to kill tissues and were then cooled to room temperature. Final conductivity measurements (EC_f_) were taken after equilibrating to 20 °C to determine maximum leakage. Membrane injury was then calculated using the following equation [46]:

(2) $$Membrane\;injury=\frac{{EC}_{0}-{EC}_{w}}{{EC}_{f}-{EC}_{w}}\times 100$$

LT50 values were calculated from both chlorophyll fluorescence and membrane injury by fitting a sigmoidal curve to freeze test changes for each branch. From those sigmoidal curves, LT50-MI was the temperature at which balsam fir would have 50% membrane injury, and LT50-CG was the temperature at which balsam fir would lose 50% of its initial chlorophyll fluorescence.

### 4.5. Lipid Extraction and Analysis

All lipids were extracted at the Kansas Lipidomic Research Center (Manhattan, KS, USA) using an extraction protocol for *Arabidopsis* leaf tissue adapted from [47]. Approximately 1 g of frozen needles were cut into smaller pieces and incubated in 1.0 mL of isopropanol with 0.01% butylated hydroxytoluene (BHT) at 75 °C for 15 min. Afterwards, 1.5 mL of chloroform and 0.6 mL of water were added to the solution. The solvent was shaken at room temperature for 1h and then transferred to a new glass tube with a Teflon-lined screw-cap using a Pasteur pipette. A total of 0.7 mL chloroform:methanol (2:1) was added, shaken for 30 min. The extraction was completed by adding 4 mL of chloroform: methanol (2:1) ten times, shaking for 1 h and collecting the solvent. The solvent extracts were washed once with 1mL KCl (1.0 M) and once with 0.66 mL water. The solvent was evaporated under nitrogen and the lipid extract was quantified and dissolved in 1.0 mL chloroform. The tissues, after lipid extraction, were dried in an oven at 105 °C and dry weights were determined (3–20 mg).

Lipid extracts were analyzed on a triple quadrupole mass spectrometer equipped for electrospray ionization (ESI–MS/MS; Applied Biosystems API 4000, Waltham, MA, USA). Acquisition and ESI-MS/MS analysis parameters are shown in Table 4 and Table 5, respectively. The lipids in each class were quantified in comparison to two internal standards of the class. Lipid species within each head group were identified by total carbon number and total double bonds. The molecular species of each head class were quantified by comparrison with the signals of the internal standards [25].

Average coefficient of variation (CoV) for lipid analytes is a function of corrections used in data processing. CoV is equal to the standard deviation of the measurements for each analyte divided by the average. CoV was calculated from the quality check samples, without any correction, using only the linear trend correction within each day’s sample set, only the correction to the overall average across sample sets, or both corrections (as conducted on the experimental data).

### 4.6. Statistical Analysis

Freeze tests with membrane injury and chlorophyll fluorescence were analyzed using an analysis of variance in Minitab 19 software (Minitab, LLC., Pennsylvania State College, PA). Temperature (5 levels) and collection month (4 levels) were used an explanatory variable, and genotype (4 levels) was used as a blocking factor. Analysis of LT50 values, NRD, and lipids was also conducted using an analysis of variance with collection month as the only explanatory variable and genotype as the blocking factor. The relationships between response variables were assessed using linear regression. Genotype was included as a blocking variable in a regression model when it accounted for a significant amount of variation. Statistical assumption of normality, homogeneity, and independence was verified for each analysis.

## 5. Conclusions

This experiment was able to address each of the original objectives. Cold acclimation was confirmed in balsam fir, with trees able to tolerate colder temperatures throughout autumn assessed via decreased membrane injury and maintenance of chlorophyll fluorescence. LT50 values decreased from approximately −23 °C in September to −46 °C in December. Polar lipid distribution changed during cold acclimation, with a significant shift from MGDG to DGDG and from GLs to PLs. The highest increase in PLs was observed in PC, though most other PLs also increased significantly. Changes in lipids were significantly related to cold acclimation.

Needle retention improved throughout cold acclimation. Needle retention was also significantly related to cold tolerance and MGDG:DGDG. In general, higher cold acclimation or a shift from MDGD to DGDG also increased needle retention. Overall, it was concluded the cold acclimation is beneficial to needle retention, possibly through increased stability of membranes due to a relative increase in DGDG. The mechanism through which cold acclimation affects needle retention requires further work, and factors beyond polar lipid shifts should also be considered moving forward.

## Figures and Tables

**Table 1:** Comparison of lipid classes by percentage of total lipids for balsam fir branches harvested at five different months. Values are expressed as the mean ± standard error as calculated from 20 replicates. The *p*-value denotes whether there was a significant difference in at least one of the sampling dates for each class of lipids. DGDG, digalactosyldiacylglycerol; LPG, lysophosphatidylglycerol; LPE, lysophosphatidylethanolamine; LPC, lysophosphatidylcholine; MGDG, monogalactosyldiacylglycerol; PA, phosphatidic acid; PC, phosphatidycholine; PE: phoshatidylethanolamine; PG, phosphatidylglycerol; PI, phosphainositol.

Lipid Class	September.	October	November	December	*p*-Value
DGDG	26.87 ± 0.36	27.10 ± 0.43	28.33 ± 0.38	30.04 ± 0.37	<0.001
MGDG	45.90 ± 0.87	40.98 ± 1.17	38.37 ± 0.81	33.86 ± 0.58	<0.001
PC	15.01 ± 0.39	18.12 ± 0.61	18.48 ± 0.44	19.56 ± 0.40	<0.001
PG	4.04 ± 0.20	3.89 ± 0.16	3.81 ± 0.15	5.11 ± 0.15	<0.001
PE	2.62 ± 0.17	3.74 ± 0.18	3.75 ± 0.21	4.76 ± 0.24	<0.001
PI	3.86 ± 0.15	3.99 ± 0.16	4.64 ± 0.17	4.75 ± 0.11	<0.001
PA	0.47 ± 0.06	0.93 ± 0.09	1.19 ± 0.34	0.66 ± 0.05	=0.049
LPC	0.05 ± 0.01	0.12 ± 0.02	0.14 ± 0.01	0.09 ± 0.01	=0.041
LPG	1.05 ± 0.19	0.96 ± 0.15	1.14 ± 0.15	1.00 ± 0.20	=0.344
LPE	0.11 ± 0.01	0.15 ± 0.01	0.14 ± 0.01	0.14 ± 0.01	=0.071

**Table 2:** Strength, significance, and linear equation of relationships between relative polar lipid concentration and LT50 values. LT50 were estimated from membrane injury (LT50-MI) and chlorophyll fluorescence (LT50-CF) regression calculated from 80 data points using LT50 as the dependent variable and relative lipid concentration as the independent variable. DGDG, digalactosyldiacylglycerol; LPG, lysophosphatidylglycerol; LPE, lysophosphatidylethanolamine; LPC, lysophosphatidylcholine; MGDG, monogalactosyldiacylglycerol; PA, phosphatidic acid; PC, phosphatidycholine; PE: phoshatidylethanolamine; PG, phosphatidylglycerol; PI, phosphainositol.

Lipid Class	LT50-MI				LT50-CF			
	R^2^ (%)	*p*-Value	Slope	Constant	R^2^ (%)	*p*-Value	Slope	Constant
DGDG	31.5	<0.001	−2.23	25.8	29.5	<0.001	−1.87	21.3
MGDG	55.0	<0.001	1.10	−80.8	42.7	<0.001	0.86	−65.6
PC	36.7	<0.001	−1.86	−3.80	26.0	<0.001	−1.36	−7.01
PG	15.2	=0.001	−3.86	−20.6	16.6	=0.008	−3.43	−16.8
PE	35.4	<0.001	−4.40	−20.6	27.7	<0.001	−3.41	−18.6
PI	30.0	<0.001	−6.13	−10.5	22.9	<0.001	−4.56	−11.6
PA	2.5	=0.180	−1.65	−35.5	3.4	=0.411	−0.73	−30.7
LPC	5.1	=0.060	−29.0	−34.0	7.8	=0.193	24.2	−28.85
LPG	0.7	=0.498	−0.69	−36.2	3.4	=0.399	−1.10	−30.1
LPE	3.1	=0.130	−30.3	−32.8	4.6	=0.208	−28.4	−27.5

**Table 3:** Temperature parameters 30 days prior to first sampling periods and between each sampling time. Temperature-related parameters are CDD (cold degree days), T_min_ (minimum temperature in the days prior to testing), T_max_ (highest temperature experienced in the month prior and since the last sampling date), and cumulative days below 0 °C. Photoperiod represents the daylight hours on the day of sampling.

Sampling Date	CDD (Days)	T_min_ (°C)	T_max_ (°C)	Days Below 0 °C	Photoperiod (h)
18 September	0	5	21	0	12.4
28 October	9	−4	18	7	10.3
25 November	102	−8	17	28	9.2
30 December	354	−23	8.3	60	8.8

**Table 4:** Acquisition parameters of lipid classes using Applied Biosystems API 4000 triple quadrupole mass spectrometer.

Parameter	PA	PC/LPC	PE/LPE	PG	PI	PS	DGDG	MGDG
Typical Scan Time (min)	3.51	1.28	3.34	3.21	4.00	4.01	1.67	1.67
Depolarization Potential (V)	100	100	100	100	100	100	90	90
Exit Potential (V)	14	14	14	14	14	14	10	10
Collision Energy (V)	25	40	28	20	25	26	24	21
Collision Exit Potential (V)	14	14	14	14	14	14	23	23

**Table 5:** ESI-MS/MS analysis parameters (using Applied Biosystems API 4000) for plant lipids. DGDG, digalactosyldiacylglycerol; ESI-MS/MS, electrospray ionization tandem mass spectrometry; MGDG, monogalactosyldiacylglycerol; PA, phosphatidic acid; PC, phosphatidylcholine; LPC, lyso- phosphatidylcholine; PE, phosphatidylethanolamine; LPE, lysophosphatidylethanolamine; PG, phosphatidylglycerol; LPG, lysophosphatidylglycerol; PI, phosphatidylinositol; PS, phosphatidylserine.

Class	Ion Analyzed	Positive Ion Scan Mode	*m/z* Range	Reference
PA	(M + NH_4_)^−^	NL of 115.00	500–850	[48]
PC/LPC	(M + H)^−^	Pre of *m/z* 184.07	450–960	[49]
PE/LPE	(M + H)^−^	NL of 141.02	420–920	[49]
PG	(M + NH_4_)^−^	NL of 189.04	650–1000	[50]
LPG	(M-H)^−^	Prec 153	-	[25]
PI	(M + NH_4_)^−^	NL of 277.06	790–950	[50]
PS	(M + H)^−^	NL of 185.01	600–920	[49]
DGDG	(M + NH_4_)^−^	NL of 341.13	890–1050	[51]
MGDG	(M + NH_4_)	NL of 179.08	700–900	[51]

## Data Availability

Data are contained within the article.
